# Warning against a Prescription Fraud

**Published:** 1913-12

**Authors:** 


### Warning Against a Prescription Fraud.

WASHINGTON, D. C.

The Department of Agriculture, under the Food and Drugs Act, has recently been investigating a new trick of certain patent medicine and proprietary medicine vendors, which it is believed is deceiving a large number of people into spending money for patent medicines under the impression that they are getting regular physicians' prescriptions for nothing.

In a number of publications the Department finds advertisements are appearing which state that the man or woman whose name is attached was saved from death from one of a number of serious diseases through some wonderful prescription given to him or her by a regular physician of unusual skill who will not allow his name to be used because of medical ethics. The advertisement states that the writer feels it to be a duty to communicate this invaluable recipe to humanity in order to save them from similar ills. The offer is then made to supply this prescription without charge to anyone who will address a post card to the advertiser. People who do not stop to wonder who is to pay for the advertisement and the return postage and writing of the prescription are caught by this fraud and ask for the prescription. In due course a regular prescription is returned. This contains a number of ordinary ingredients and then under a technical name will call for a large proportion of some patent medicine or proprietary drug. The recipient takes this to a drug store to be filled and the druggist finds that he has to buy some of this patent preparation in order to fill it. He, therefore, has to order a large package or bottle of it and to make a profit must charge the customer a good, stiff price for filling the prescription. The customer, of course, gets what is in effect simply a patent medicine which, save that it bears a druggist's label and a prescription number, is the same as a patent medicine sold under the maker's own label and in the maker's own bottle.

The Government cannot reach these people under either the Food and Drugs Act or the Postal Laws, because the scheme is so planned as to evade Government laws. The deception and misrepresentation appears in advertisements, circulars, letters, etc., separate from the package and the medicines are seldom sent through the mails. The best the Department can do, therefore, is to warn the people to be particularly suspicious of those who spend money for advertising space, postage, and letter writing, seemingly out of their love for humanity. In all of these cases there is a profit-making scheme back of the seeming philanthropy.

